# Dengue infections in travellers

**DOI:** 10.1179/2046904712Z.00000000050

**Published:** 2012-05

**Authors:** Annelies Wilder-Smith

**Affiliations:** Institute of Public Health, University of Heidelberg, Germany

**Keywords:** Dengue, Travellers, GeoSentinel, Autochthonous spread, Sentinel surveillance

## Abstract

Dengue has been designated a major international public health problem by the World Health Organization (WHO). It is endemic in most tropical and sub-tropical countries, which are also popular tourist destinations. Travellers are not only at significant risk of acquiring dengue but they also contribute to its spread to non-endemic regions. Furthermore, they may serve as sentinels to alert the international community to epidemics in dengue-endemic regions. GeoSentinel, a global surveillance network, monitors all travel-related illnesses and estimates that dengue accounts for 2% of all illness in travellers returning from dengue-endemic regions. In fact, in travellers returning from South-east Asia, dengue is now a more frequent cause of febrile illness than malaria. Dengue-infected travellers returning home to countries where the vector exists can place the local population at risk of further spread of the disease with subsequent autochthonous cycles of infection. The true incidence of dengue amongst travellers may be underestimated because of variability in reporting requirements in different countries and under-diagnosis owing to the non-specific clinical presentation of the disease. Risk factors for acquiring dengue include duration of stay, season of travel and epidemic activity at the destination. Any pre-travel advice on the risks of developing dengue infections should consider these factors.

## Introduction

**Figure 1:**
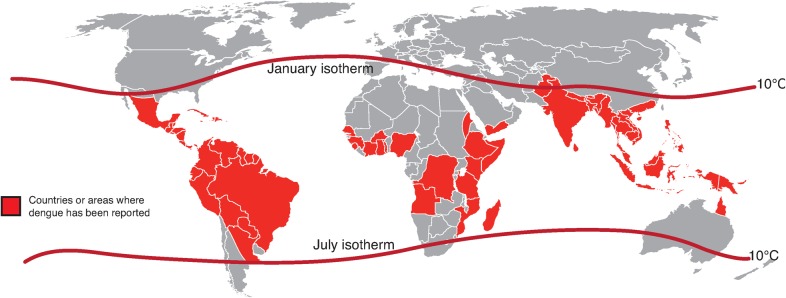
Regions at risk of dengue transmission in 2010, as indicated by the contour lines of the January and July isotherms, which define the geographical limits between which *Aedes aegypti* survives year-round^2^

Dengue is endemic in most tropical and sub-tropical countries, and has been designated a major international public health concern by the World Health Organization (WHO) (Fig. 1).1,^2^ Many countries in dengue-endemic regions are also popular tourist destinations, and the rise in international travel to these regions has played a significant role in the global spread of the disease.^3^ With forecasts of international tourist arrivals predicted to reach 1·8 billion by 2030, increasingly involving emerging growth markets in Asia and Latin America,^4^ the potential for dengue to expand to areas currently free of the disease is significant.

Travellers are at significant risk of acquiring the disease and also contribute to its spread to non-endemic regions.^5^ They may further serve as sentinels to alert the international community to epidemics in dengue-endemic regions and to the spread of dengue virus serotypes and genotypes.^6^ This article discusses the impact of travel in the epidemiology of dengue infections.

## Epidemiology and Risk of Travel-Related Dengue

An estimated 50 million dengue infections occur every year, with approximately 2·5 billion people living at risk of infection in endemic regions.^6^ There has been a 30-fold increase in the incidence of dengue over the past 50 years, with spread to new regions; and international travel is increasingly a contributory factor.^6^

GeoSentinel, a data-collection network that monitors all travel-related illnesses across 54 clinics globally, has estimated that dengue accounts for 2% of all illness in travellers returning from dengue-endemic regions.^7^ A study found that, between 1997 and 2006, dengue was imported most commonly from South-east Asia (51%), followed by South Central Asia (17%), Latin America (15%), the Caribbean (9%), parts of Africa (5%) and Oceania (2%).^7^

The proportion of febrile travellers returning from tropical and sub-tropical countries being diagnosed with dengue has increased from 2% in the early 1990s to 16% by 2005.^3^ Dengue is now a more frequent cause of febrile illness than malaria in travellers returning from South-east Asia.^8^

Prospective seroconversion studies of travellers to endemic countries estimated an incidence of 2·9% in Dutch travellers who spent approximately 1 month in Asia^9^ and 6·7% in Israelis who travelled to tropical countries for approximately 6 months.^10^ However, the true incidence of dengue in travellers may be underestimated because of variability in reporting requirements in different countries and under-diagnosis owing to the non-specific clinical presentation of the disease.^11^

## Spread of Dengue to New Regions

Dengue-infected travellers returning home can place the local population at risk of further spread of the disease wherever the mosquito vectors, *Aedes aegypti* and/or *A. albopictus*, the primary and secondary vectors, respectively, are present.^6^ The increasing global spread of the vectors means that many non-endemic countries harbour populations of mosquitoes capable of spreading the dengue virus introduced by infected returning travellers.^3^

In Europe, for example, imported cases of dengue have been reported to have risen from 64 in 1999 to 224 in 2002, although the number of reported cases has subsequently stabilised.^12^ Dengue infections occur commonly in US citizens returning from endemic areas and are more prevalent than malaria among those returning from the Caribbean, South America, South Central Asia and South-east Asia.^13^ Australia has also seen a dramatic rise in the number of dengue cases in returned travellers, particularly those who have visited South-east Asia, with an increase of approximately 350% in the number of reported dengue cases between 2004 and 2007 and 2008 and 2011.14,^15^

Following the return from dengue-endemic countries of infected travellers, autochthonous cycles of infection can subsequently be established.^3^ Locally acquired dengue infections have been reported in Europe,16,^17^ the United States (US)^18^ and Australia.^19^

Populations in non-endemic countries may also be at risk of acquiring dengue by other means. Although representing only a small proportion of dengue cases, the disease can also be spread by mechanisms not involving mosquitoes as vectors, such as hospital-acquired transmission mainly through blood transfusion. Dengue transmission via needle-stick injury^20^ or mucocutaneous exposure to blood^21^ has been reported in healthcare workers in non-endemic countries. However, blood products are not screened for dengue, and further studies are needed to assess the risk of infected blood for transmission.^22^

## Travellers as Sentinels

Travellers may also play an important role as sentinels in alerting the international community to the onset of epidemics in endemic regions where surveillance is often poor.6,^23^

During a 2002 epidemic in South-east Asia, for example, GeoSentinel provided an international alert by publicising an increase in travel-related dengue originating from Thailand,^24^ before official Thai surveillance data became available. Furthermore, analysis of the 1998 travel-related disease pattern of infection from the GeoSentinel database predicted the 2002 epidemic.^7^

## Seasonality and Trends in Dengue Infections

Risk factors for acquiring dengue include duration of stay, season of travel and epidemic activity at the destination.^7^–^9^ Although reports of dengue cases increase in the rainy season, this varies according to country and even between regions within countries. It is therefore difficult to definitively correlate rainfall with the incidence of dengue.^7^

**Figure 2:**
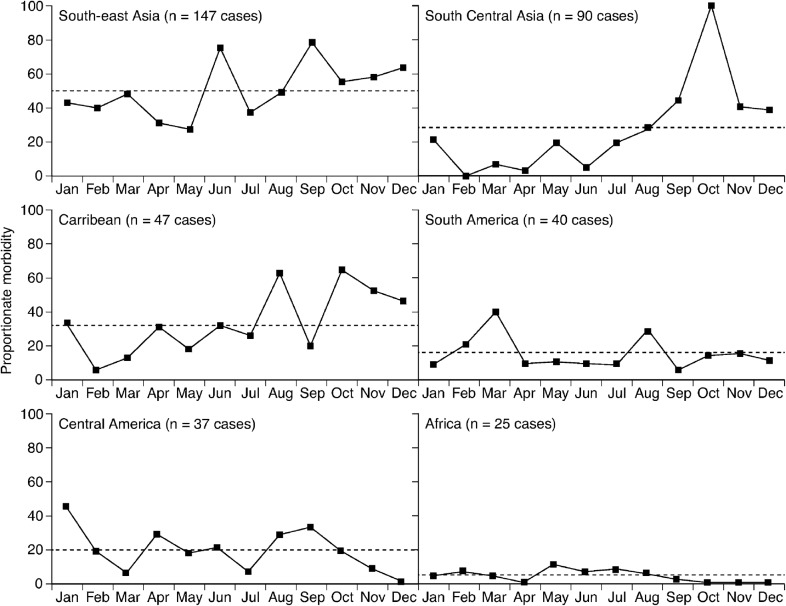
Seasonality of dengue in returned travellers according to region. The dashed lines represent the mean proportionate morbidity (the number of dengue cases per 1000 ill returned travellers) in travellers for all months for the specified region during 1997–2006^7^

The GeoSentinel study of cases between 1997 and 2006 examined the seasonality of dengue (Fig. 2).^7^ Seasonal patterns were observed during the study period in Asia, the Caribbean and South America, but were not as strong in Central America and Africa. However, the study demonstrated a difference in seasonality between outbreak and non-outbreak years. For South-east Asia, for example, there were peaks in dengue cases in June and September in non-epidemic years, while during epidemics excess cases were recorded for almost every month, particularly during April to August. Results of the study therefore suggest that any pre-travel advice on the risks of developing dengue infections should consider epidemic activity and seasonal patterns.

Mathematical models take these risk factors into account and may be useful tools in providing evidence-based estimates of the risk of dengue transmission in travellers to dengue-endemic regions. For example, such models estimated that a non-immune traveller staying in Singapore for 1 week during the high season in 2005 had a 0·17% risk of acquiring dengue.^25^

## Characteristics of Travel-Related Dengue

A large proportion of cases of travel-related dengue, as in endemic populations, are asymptomatic or minimally symptomatic.9,^26^ However, when symptoms do develop, because of their non-specific nature, they are often misdiagnosed as some other febrile illness such as chikungunya, malaria, typhoid fever and rickettsial infection.^23^ Furthermore, as laboratory-based diagnosis is often unavailable at the time of care, diagnosis frequently has to be made solely on the clinical presentation.^3^ However, in patients with febrile illness, life-threatening but potentially treatable diseases such as typhoid fever and malaria should always be excluded first. Also, given the short incubation period, a diagnosis of dengue is unlikely if the initial presentation is more than 2 weeks after return from an endemic country.^3^

Dengue disease is considered to occur as a continuous spectrum of severity.^27^ The current WHO case definition for diagnosis of dengue is separated into patients with severe and non-severe dengue, with the large group of those with non-severe dengue being sub-divided into patients with and without warning signs.^6^ Surveillance reports from the European Network on Imported Infectious Disease Surveillance (TropNetEurop) showed that European travellers present with a wide variety of symptoms, but the majority with a confirmed or probable diagnosis of dengue presented with uncomplicated dengue with the typical symptoms of fever, headache, fatigue and musculoskeletal pain.^22^

However, certain combinations of clinical features and laboratory abnormalities may be better able to predict dengue in travellers. In a study of ill returned Australian travellers, a diagnosis of dengue was 18-, 71-, and 230-times more likely if the combinations of fever and leucopenia, fever and rash, and fever, rash and leucopenia, respectively, were present.^14^ Owing to the increasing prevalence and non-specific symptoms of dengue, it is important that healthcare professionals across the world be familiar with its clinical features.

## Dengue *vs* Malaria

The GeoSentinel study described above compared traveller characteristics in patients with dengue and malaria.^7^ Dengue affected both sexes equally, unlike malaria, which affected male travellers more frequently than female travellers. Duration of travel was slightly shorter for travellers with dengue who visited as tourists than for those with malaria who predominantly visited friends or relatives.

## Severe Dengue

According to the WHO case definition, severe dengue, encompassing the symptoms of dengue haemorrhagic fever (DHF), is characterised by severe plasma leakage, haemorrhage and organ impairment.^6^ Severe dengue appears to be less common in travellers than in populations in endemic countries.^12^ In endemic areas, approximately 6% of symptomatic dengue cases progress to DHF.^28^ In comparison, intensified surveillance in travellers performed within TropNetEurop revealed that, of 219 dengue-infected travellers, 0·9% fulfilled the 1997 WHO criteria for DHF,^29^ although 11% of patients experienced severe clinical manifestations.^30^

Secondary dengue infection is considered to be a significant risk factor for DHF^31^ as it is thought that non-neutralising cross-reacting antibodies from the primary infection enhance the infecting ability of virus particles.^32^ Given their lack of previous exposure, travellers are unlikely to have pre-existing antibodies to dengue.

Another contributory factor to the lower incidence of DHF in travellers is that the large majority are adults^33^ who are reported to have a lower risk of DHF than children.^34^

## Dengue in Children

Children represent a significant proportion of the travelling public, accounting for 7% (1·9 million) of travellers living in the US.^33^ Classic and severe dengue in children pose a significant burden on endemic countries such as Thailand, which has a mean annual burden attributable to dengue of 465·3 disability-adjusted life-years over 5 years.^35^

A study of over 1500 ill paediatric travellers reporting to GeoSentinel clinics in 19 countries identified dengue and typhoid fever as the most frequent causes of systemic febrile illness in children returning from tropical regions other than sub-Saharan Africa.^33^

**Figure 3:**
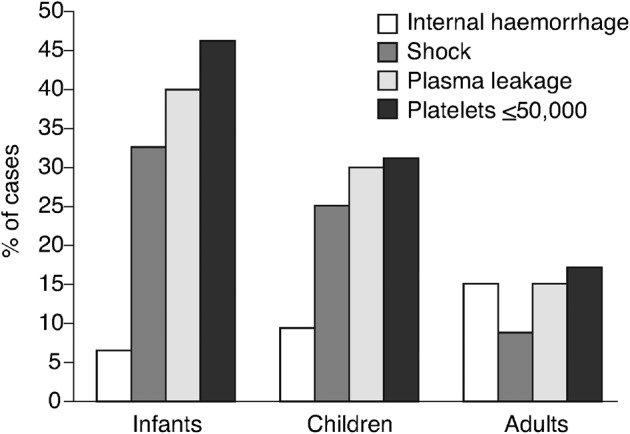
Prevalence of severe dengue symptoms (internal haemorrhage, shock, signs of plasma leakage and/or marked thrombocytopenia) in infants, children and adults^34^

Children have a higher risk than adults of developing severe dengue,34,^36^ a leading cause of morbidity and death in this age-group (Fig. 3).^34^ The risk of mortality from a secondary infection is nearly 15-fold higher than in adults.^36^ It is believed that 10% of children with secondary infection go on to develop DHF.^31^

## Protection for Travellers Against Dengue

There is currently no licensed dengue vaccine, and measures such as vector control are proving inadequate in reducing the incidence of the disease.37,^38^ Therefore, with only supportive treatment of dengue available, protection against dengue is limited to avoidance of mosquito bites with the use of insect repellents, protective clothing and insecticides.^39^ Avoidance of litter and containers with stagnant water is also advised.^39^ Protective measures need to be taken during the day as this is when mosquitoes bite, with only limited effectiveness of night-time measures such as insecticide-treated bed-nets.^3^ An effective and cost-effective vaccine against dengue would therefore be a major advance in controlling the disease.28,^38^ Given the high incidence of the disease in travellers, a vaccine for them may also be indicated, provided that it is safe, convenient to administer and affordable.^40^ The vaccine candidate furthest in development is a chimeric vaccine by Sanofi Pasteur. With the lead candidate vaccine showing encouraging results in late-stage clinical trials, the outlook for introduction of a vaccine against all four dengue serotypes into national immunisation programmes of endemic countries is promising.^41^

## Conclusion

The incidence of dengue in international travellers, including children, is rising. Furthermore, travellers contribute to the geographic spread of dengue and its introduction to previously uninfected areas. The rising numbers of dengue cases reported worldwide, and identification of locally acquired dengue infections in non-endemic regions, emphasise the need for surveillance of travellers returning from endemic areas. Since the incidence of dengue demonstrates seasonality and variation according to destination of travel, pre-travel advice should take into account epidemic activity, seasonal patterns and travel destination.
